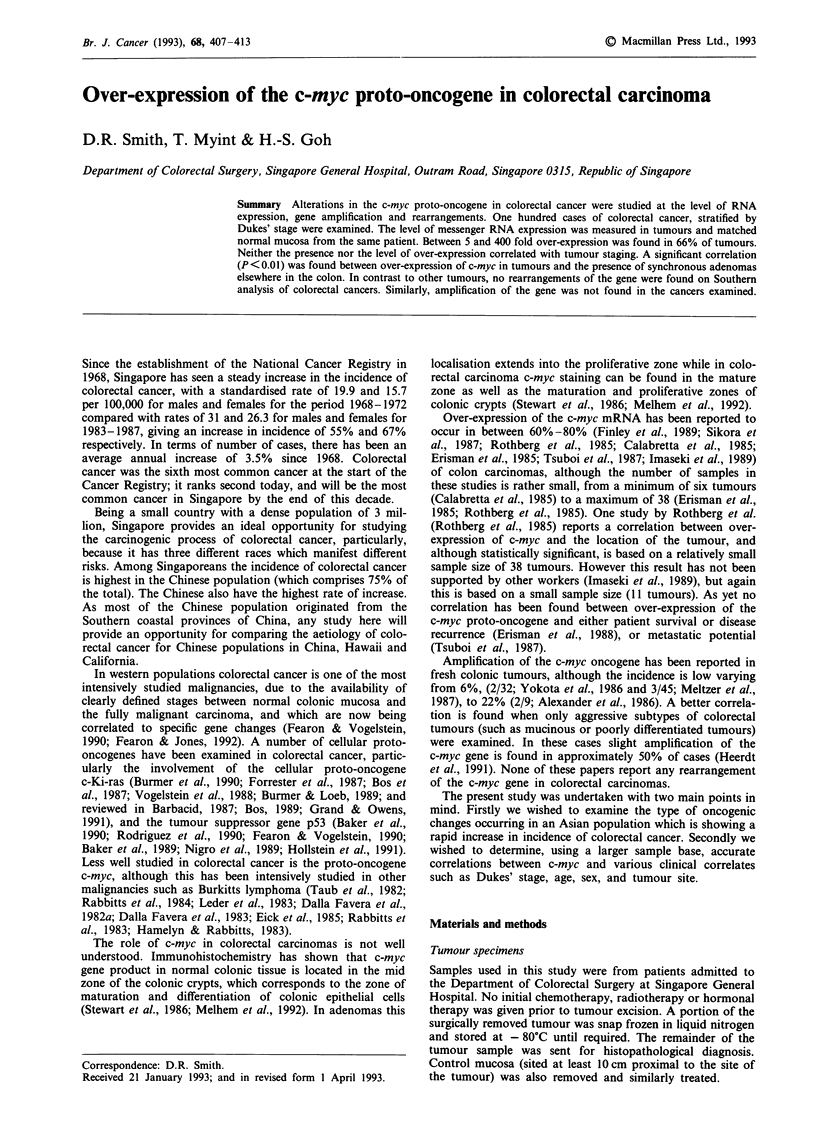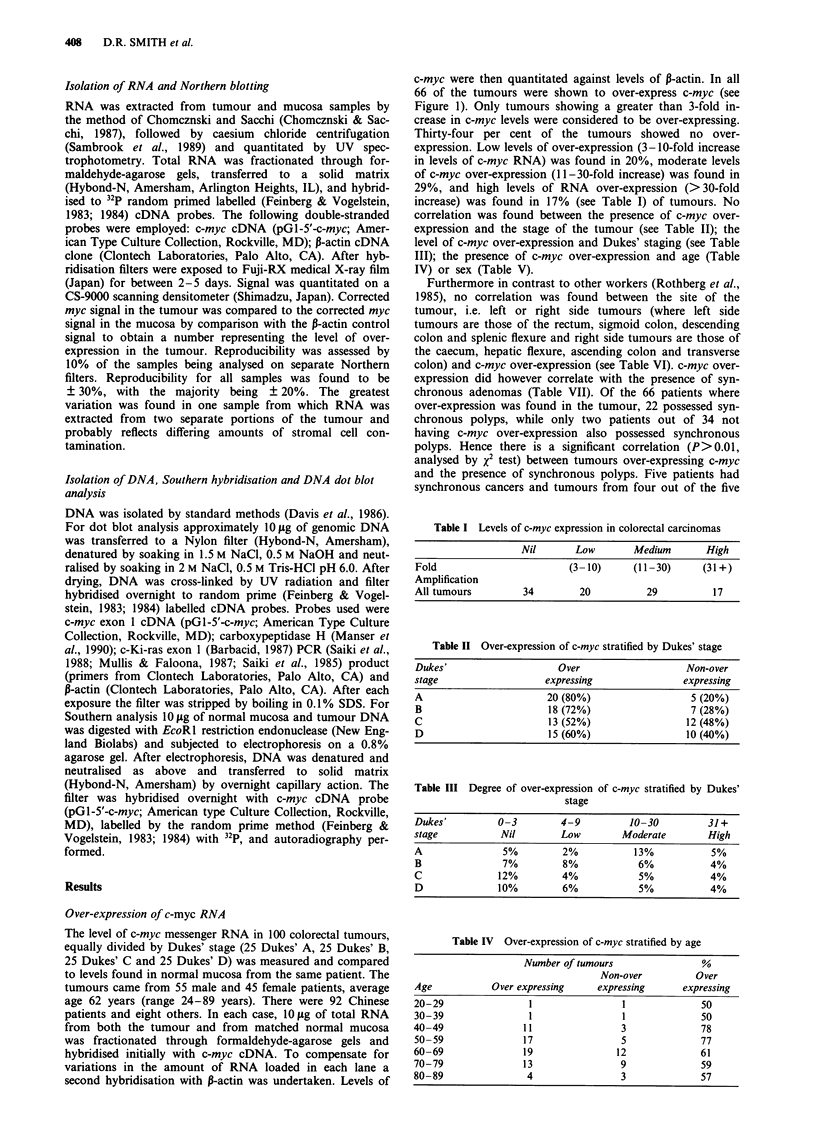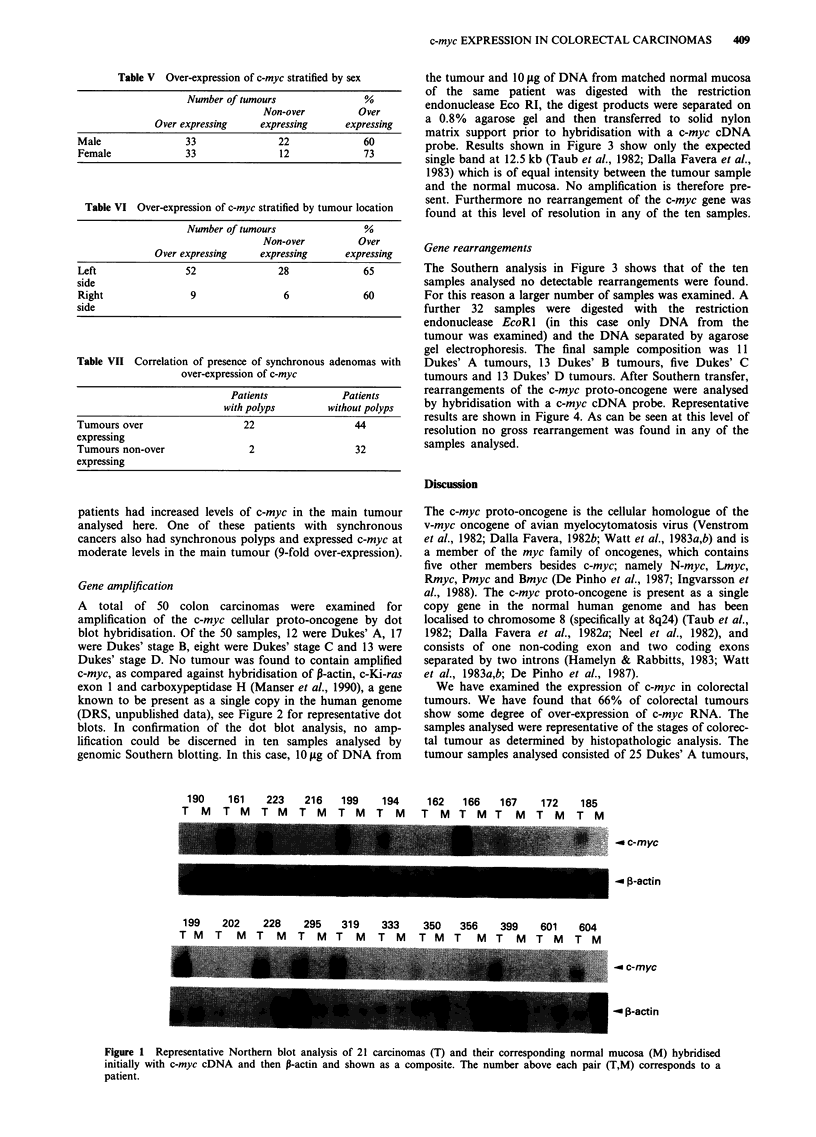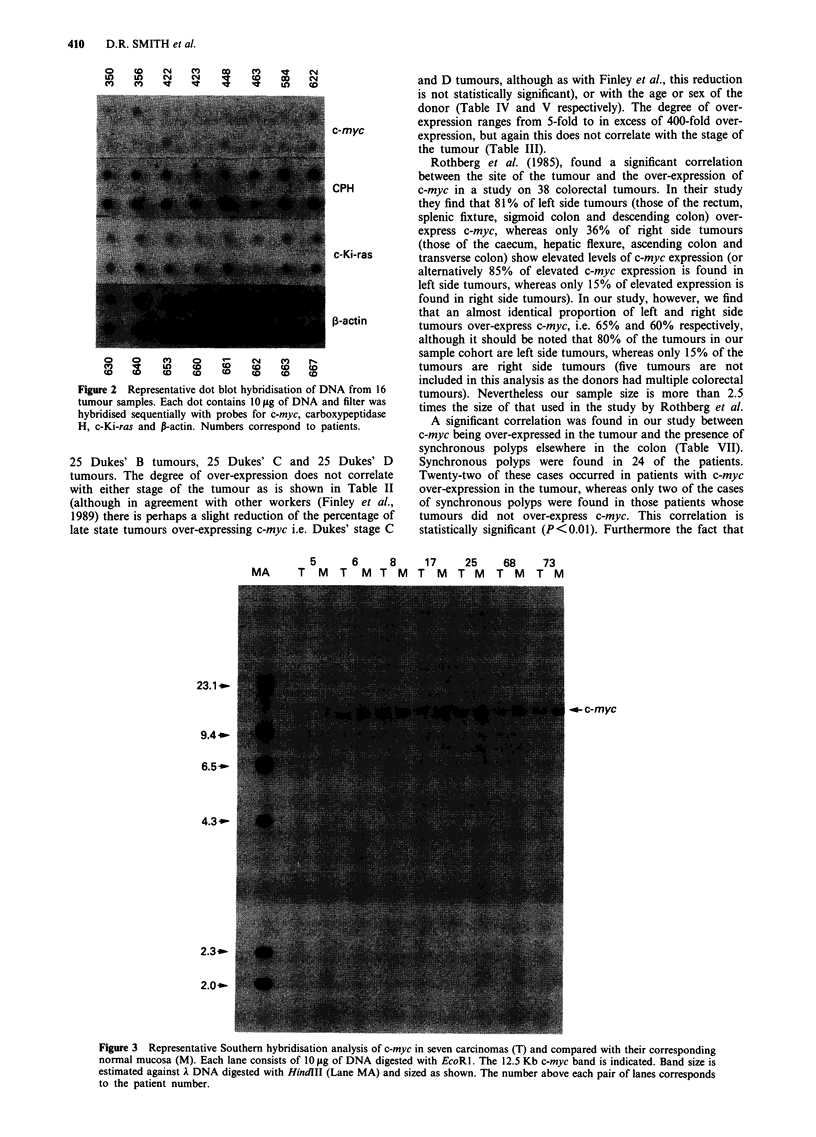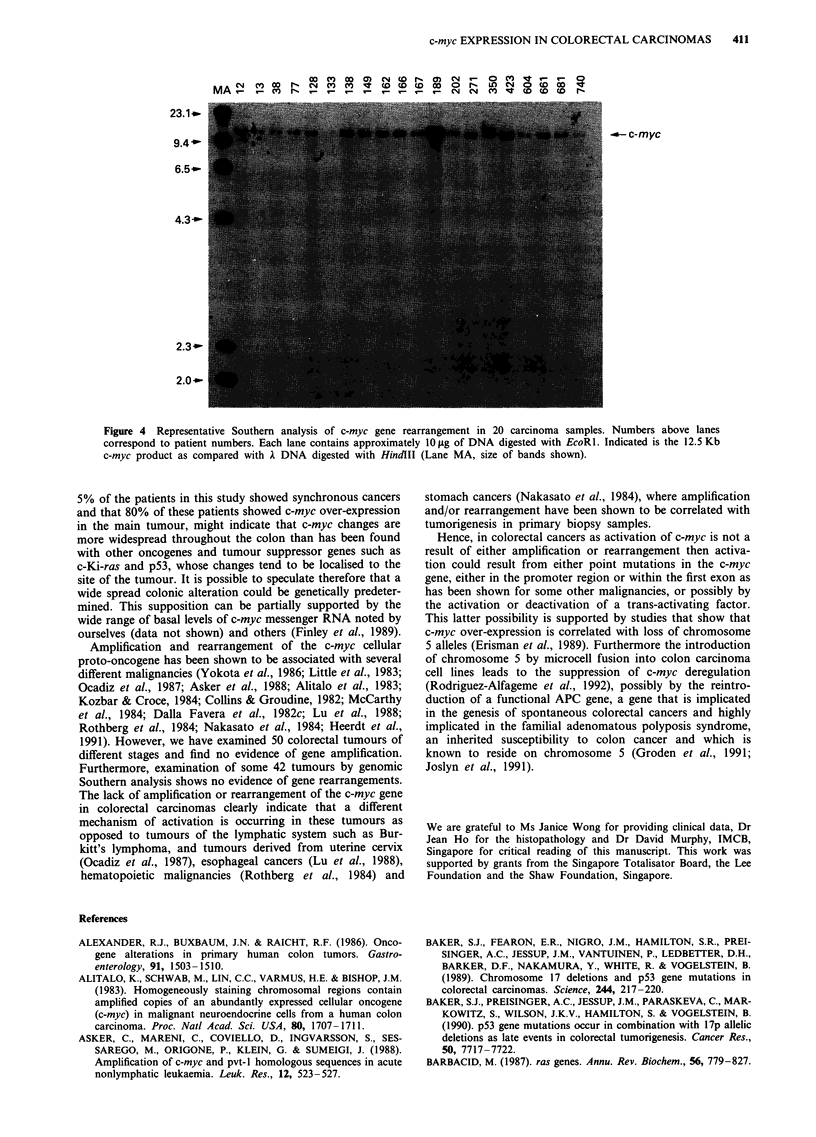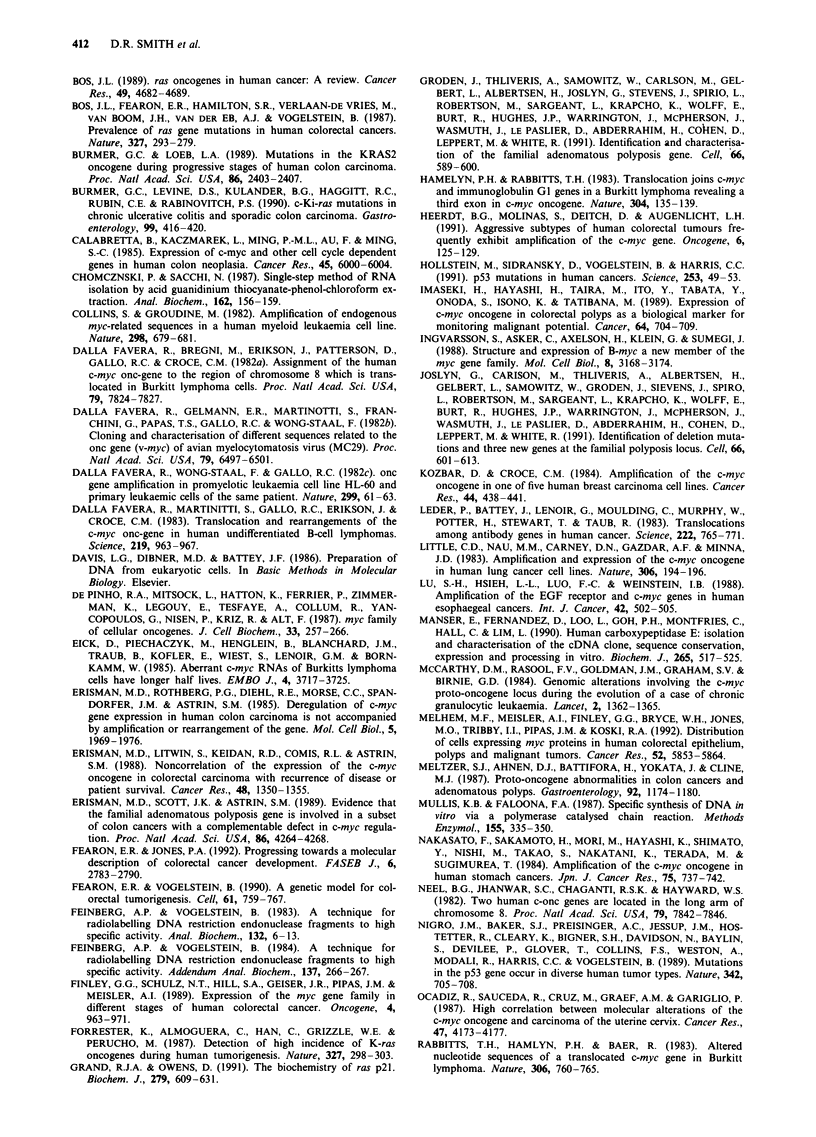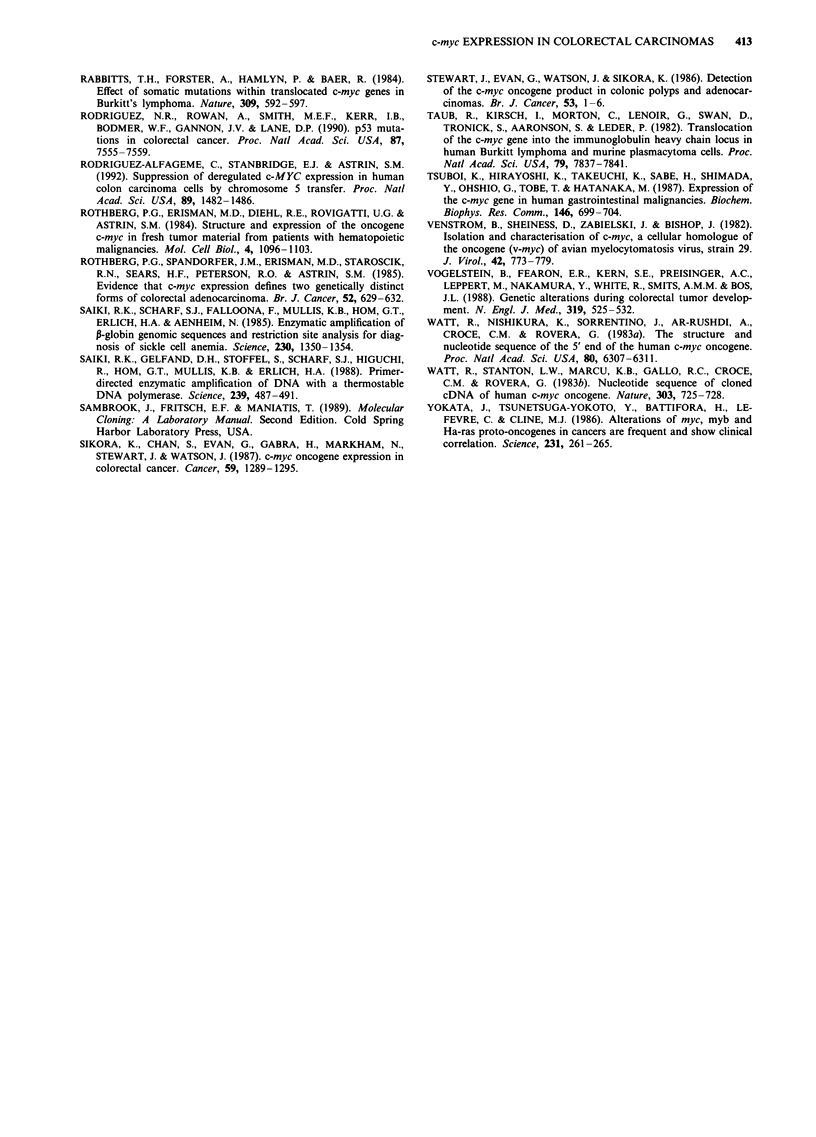# Over-expression of the c-myc proto-oncogene in colorectal carcinoma.

**DOI:** 10.1038/bjc.1993.350

**Published:** 1993-08

**Authors:** D. R. Smith, T. Myint, H. S. Goh

**Affiliations:** Department of Colorectal Surgery, Singapore General Hospital.

## Abstract

**Images:**


*Br. J. Cancer* (1993), **68**, 407–413 © Macmillan Press Ltd., 1993

### Over-expression of the c-*myc* proto-oncogene in colorectal carcinoma

D.R. Smith, T. Myint & H.-S. Goh

*Department of Colorectal Surgery, Singapore General Hospital, Outram Road, Singapore 0315, Republic of Singapore*

**Summary** Alterations in the c-*myc* proto-oncogene in colorectal cancer were studied at the level of RNA expression, gene amplification and rearrangements. One hundred cases of colorectal cancer, stratified by Dukes' stage were examined. The level of messenger RNA expression was measured in tumours and matched normal mucosa from the same patient. Between 5 and 400 fold over-expression was found in 66% of tumours. Neither the presence nor the level of over-expression correlated with tumour staging. A significant correlation ($P < 0.01$) was found between over-expression of c-*myc* in tumours and the presence of synchronous adenomas elsewhere in the colon. In contrast to other tumours, no rearrangements of the gene were found on Southern analysis of colorectal cancers. Similarly, amplification of the gene was not found in the cancers examined.

Since the establishment of the National Cancer Registry in 1968, Singapore has seen a steady increase in the incidence of colorectal cancer, with a standardised rate of 19.9 and 15.7 per 100,000 for males and females for the period 1968–1972 compared with rates of 31 and 26.3 for males and females for 1983–1987, giving an increase in incidence of 55% and 67% respectively. In terms of number of cases, there has been an average annual increase of 3.5% since 1968. Colorectal cancer was the sixth most common cancer at the start of the Cancer Registry; it ranks second today, and will be the most common cancer in Singapore by the end of this decade.

Being a small country with a dense population of 3 million, Singapore provides an ideal opportunity for studying the carcinogenic process of colorectal cancer, particularly, because it has three different races which manifest different risks. Among Singaporeans the incidence of colorectal cancer is highest in the Chinese population (which comprises 75% of the total). The Chinese also have the highest rate of increase. As most of the Chinese population originated from the Southern coastal provinces of China, any study here will provide an opportunity for comparing the aetiology of colorectal cancer for Chinese populations in China, Hawaii and California.

In western populations colorectal cancer is one of the most intensively studied malignancies, due to the availability of clearly defined stages between normal colonic mucosa and the fully malignant carcinoma, and which are now being correlated to specific gene changes (Fearon & Vogelstein, 1990; Fearon & Jones, 1992). A number of cellular proto-oncogenes have been examined in colorectal cancer, particularly the involvement of the cellular proto-oncogene c-Ki-ras (Burmer *et al.*, 1990; Forrester *et al.*, 1987; Bos *et al.*, 1987; Vogelstein *et al.*, 1988; Burmer & Loeb, 1989; and reviewed in Barbacid, 1987; Bos, 1989; Grand & Owens, 1991), and the tumour suppressor gene p53 (Baker *et al.*, 1990; Rodriguez *et al.*, 1990; Fearon & Vogelstein, 1990; Baker *et al.*, 1989; Nigro *et al.*, 1989; Hollstein *et al.*, 1991). Less well studied in colorectal cancer is the proto-oncogene c-*myc*, although this has been intensively studied in other malignancies such as Burkitts lymphoma (Taub *et al.*, 1982; Rabbitts *et al.*, 1984; Leder *et al.*, 1983; Dalla Favera *et al.*, 1982*a*; Dalla Favera *et al.*, 1983; Eick *et al.*, 1985; Rabbitts *et al.*, 1983; Hamelyn & Rabbitts, 1983).

The role of c-*myc* in colorectal carcinomas is not well understood. Immunohistochemistry has shown that c-*myc* gene product in normal colonic tissue is located in the mid zone of the colonic crypts, which corresponds to the zone of maturation and differentiation of colonic epithelial cells (Stewart *et al.*, 1986; Melhem *et al.*, 1992). In adenomas this localisation extends into the proliferative zone while in colorectal carcinoma c-*myc* staining can be found in the mature zone as well as the maturation and proliferative zones of colonic crypts (Stewart *et al.*, 1986; Melhem *et al.*, 1992).

Over-expression of the c-*myc* mRNA has been reported to occur in between 60%–80% (Finley *et al.*, 1989; Sikora *et al.*, 1987; Rothberg *et al.*, 1985; Calabretta *et al.*, 1985; Erisman *et al.*, 1985; Tsuboi *et al.*, 1987; Imaseki *et al.*, 1989) of colon carcinomas, although the number of samples in these studies is rather small, from a minimum of six tumours (Calabretta *et al.*, 1985) to a maximum of 38 (Erisman *et al.*, 1985; Rothberg *et al.*, 1985). One study by Rothberg *et al.* (Rothberg *et al.*, 1985) reports a correlation between over-expression of c-*myc* and the location of the tumour, and although statistically significant, is based on a relatively small sample size of 38 tumours. However this result has not been supported by other workers (Imaseki *et al.*, 1989), but again this is based on a small sample size (11 tumours). As yet no correlation has been found between over-expression of the c-*myc* proto-oncogene and either patient survival or disease recurrence (Erisman *et al.*, 1988), or metastatic potential (Tsuboi *et al.*, 1987).

Amplification of the c-*myc* oncogene has been reported in fresh colonic tumours, although the incidence is low varying from 6%, (2/32; Yokota *et al.*, 1986 and 3/45; Meltzer *et al.*, 1987), to 22% (2/9; Alexander *et al.*, 1986). A better correlation is found when only aggressive subtypes of colorectal tumours (such as mucinous or poorly differentiated tumours) were examined. In these cases slight amplification of the c-*myc* gene is found in approximately 50% of cases (Heerdt *et al.*, 1991). None of these papers report any rearrangement of the c-*myc* gene in colorectal carcinomas.

The present study was undertaken with two main points in mind. Firstly we wished to examine the type of oncogenic changes occurring in an Asian population which is showing a rapid increase in incidence of colorectal cancer. Secondly we wished to determine, using a larger sample base, accurate correlations between c-*myc* and various clinical correlates such as Dukes' stage, age, sex, and tumour site.

### Materials and methods

### *Tumour specimens*

Samples used in this study were from patients admitted to the Department of Colorectal Surgery at Singapore General Hospital. No initial chemotherapy, radiotherapy or hormonal therapy was given prior to tumour excision. A portion of the surgically removed tumour was snap frozen in liquid nitrogen and stored at $-80°C$ until required. The remainder of the tumour sample was sent for histopathological diagnosis. Control mucosa (sited at least 10 cm proximal to the site of the tumour) was also removed and similarly treated.

Correspondence: D.R. Smith.
Received 21 January 1993; and in revised form 1 April 1993.

*Isolation of RNA and Northern blotting*

RNA was extracted from tumour and mucosa samples by the method of Chomcznski and Sacchi (Chomcznski & Sacchi, 1987), followed by caesium chloride centrifugation (Sambrook *et al.*, 1989) and quantitated by UV spectrophotometry. Total RNA was fractionated through formaldehyde-agarose gels, transferred to a solid matrix (Hybond-N, Amersham, Arlington Heights, IL), and hybridised to $^{32}P$ random primed labelled (Feinberg & Vogelstein, 1983; 1984) cDNA probes. The following double-stranded probes were employed: c-*myc* cDNA (pG1-5'-c-*myc*; American Type Culture Collection, Rockville, MD); β-actin cDNA clone (Clontech Laboratories, Palo Alto, CA). After hybridisation filters were exposed to Fuji-RX medical X-ray film (Japan) for between 2–5 days. Signal was quantitated on a CS-9000 scanning densitometer (Shimadzu, Japan). Corrected *myc* signal in the tumour was compared to the corrected *myc* signal in the mucosa by comparison with the β-actin control signal to obtain a number representing the level of over-expression in the tumour. Reproducibility was assessed by 10% of the samples being analysed on separate Northern filters. Reproducibility for all samples was found to be ± 30%, with the majority being ± 20%. The greatest variation was found in one sample from which RNA was extracted from two separate portions of the tumour and probably reflects differing amounts of stromal cell contamination.

*Isolation of DNA, Southern hybridisation and DNA dot blot analysis*

DNA was isolated by standard methods (Davis *et al.*, 1986). For dot blot analysis approximately 10 μg of genomic DNA was transferred to a Nylon filter (Hybond-N, Amersham), denatured by soaking in 1.5 M NaCl, 0.5 M NaOH and neutralised by soaking in 2 M NaCl, 0.5 M Tris-HCl pH 6.0. After drying, DNA was cross-linked by UV radiation and filter hybridised overnight to random prime (Feinberg & Vogelstein, 1983; 1984) labelled cDNA probes. Probes used were c-*myc* exon 1 cDNA (pG1-5'-c-*myc*; American Type Culture Collection, Rockville, MD); carboxypeptidase H (Manser *et al.*, 1990); c-Ki-ras exon 1 (Barbacid, 1987) PCR (Saiki *et al.*, 1988; Mullis & Faloona, 1987; Saiki *et al.*, 1985) product (primers from Clontech Laboratories, Palo Alto, CA) and β-actin (Clontech Laboratories, Palo Alto, CA). After each exposure the filter was stripped by boiling in 0.1% SDS. For Southern analysis 10 μg of normal mucosa and tumour DNA was digested with *Eco*R1 restriction endonuclease (New England Biolabs) and subjected to electrophoresis on a 0.8% agarose gel. After electrophoresis, DNA was denatured and neutralised as above and transferred to solid matrix (Hybond-N, Amersham) by overnight capillary action. The filter was hybridised overnight with c-*myc* cDNA probe (pG1-5'-c-*myc*; American type Culture Collection, Rockville, MD), labelled by the random prime method (Feinberg & Vogelstein, 1983; 1984) with $^{32}P$, and autoradiography performed.

### Results

*Over-expression of c-*myc *RNA*

The level of c-*myc* messenger RNA in 100 colorectal tumours, equally divided by Dukes' stage (25 Dukes' A, 25 Dukes' B, 25 Dukes' C and 25 Dukes' D) was measured and compared to levels found in normal mucosa from the same patient. The tumours came from 55 male and 45 female patients, average age 62 years (range 24–89 years). There were 92 Chinese patients and eight others. In each case, 10 μg of total RNA from both the tumour and from matched normal mucosa was fractionated through formaldehyde-agarose gels and hybridised initially with c-*myc* cDNA. To compensate for variations in the amount of RNA loaded in each lane a second hybridisation with β-actin was undertaken. Levels of c-*myc* were then quantitated against levels of β-actin. In all 66 of the tumours were shown to over-express c-*myc* (see Figure 1). Only tumours showing a greater than 3-fold increase in c-*myc* levels were considered to be over-expressing. Thirty-four per cent of the tumours showed no over-expression. Low levels of over-expression (3–10-fold increase in levels of c-*myc* RNA) was found in 20%, moderate levels of c-*myc* over-expression (11–30-fold increase) was found in 29%, and high levels of RNA over-expression (>30-fold increase) was found in 17% (see Table I) of tumours. No correlation was found between the presence of c-*myc* over-expression and the stage of the tumour (see Table II); the level of c-*myc* over-expression and Dukes' staging (see Table III); the presence of c-*myc* over-expression and age (Table IV) or sex (Table V).

Furthermore in contrast to other workers (Rothberg *et al.*, 1985), no correlation was found between the site of the tumour, i.e. left or right side tumours (where left side tumours are those of the rectum, sigmoid colon, descending colon and splenic flexure and right side tumours are those of the caecum, hepatic flexure, ascending colon and transverse colon) and c-*myc* over-expression (see Table VI). c-*myc* over-expression did however correlate with the presence of synchronous adenomas (Table VII). Of the 66 patients where over-expression was found in the tumour, 22 possessed synchronous polyps, while only two patients out of 34 not having c-*myc* over-expression also possessed synchronous polyps. Hence there is a significant correlation ($P > 0.01$, analysed by $\chi^2$ test) between tumours over-expressing c-*myc* and the presence of synchronous polyps. Five patients had synchronous cancers and tumours from four out of the five

**Table I** Levels of c-*myc* expression in colorectal carcinomas

| | *Nil* | *Low* | *Medium* | *High* |
|---|---|---|---|---|
| Fold Amplification | | (3–10) | (11–30) | (31+) |
| All tumours | 34 | 20 | 29 | 17 |

**Table II** Over-expression of c-*myc* stratified by Dukes' stage

| *Dukes' stage* | *Over expressing* | *Non-over expressing* |
|---|---|---|
| A | 20 (80%) | 5 (20%) |
| B | 18 (72%) | 7 (28%) |
| C | 13 (52%) | 12 (48%) |
| D | 15 (60%) | 10 (40%) |

**Table III** Degree of over-expression of c-*myc* stratified by Dukes' stage

| *Dukes' stage* | *0–3 Nil* | *4–9 Low* | *10–30 Moderate* | *31+ High* |
|---|---|---|---|---|
| A | 5% | 2% | 13% | 5% |
| B | 7% | 8% | 6% | 4% |
| C | 12% | 4% | 5% | 4% |
| D | 10% | 6% | 5% | 4% |

**Table IV** Over-expression of c-*myc* stratified by age

| | *Number of tumours* | | *%* |
|---|---|---|---|
| *Age* | *Over expressing* | *Non-over expressing* | *Over expressing* |
| 20–29 | 1 | 1 | 50 |
| 30–39 | 1 | 1 | 50 |
| 40–49 | 11 | 3 | 78 |
| 50–59 | 17 | 5 | 77 |
| 60–69 | 19 | 12 | 61 |
| 70–79 | 13 | 9 | 59 |
| 80–89 | 4 | 3 | 57 |

**Table V** Over-expression of c-*myc* stratified by sex

| | *Number of tumours* | | *%* |
|---|---|---|---|
| | *Over expressing* | *Non-over expressing* | *Over expressing* |
| Male | 33 | 22 | 60 |
| Female | 33 | 12 | 73 |

**Table VI** Over-expression of c-*myc* stratified by tumour location

| | *Number of tumours* | | *%* |
|---|---|---|---|
| | *Over expressing* | *Non-over expressing* | *Over expressing* |
| Left side | 52 | 28 | 65 |
| Right side | 9 | 6 | 60 |

**Table VII** Correlation of presence of synchronous adenomas with over-expression of c-*myc*

| | *Patients with polyps* | *Patients without polyps* |
|---|---|---|
| Tumours over expressing | 22 | 44 |
| Tumours non-over expressing | 2 | 32 |

patients had increased levels of c-*myc* in the main tumour analysed here. One of these patients with synchronous cancers also had synchronous polyps and expressed c-*myc* at moderate levels in the main tumour (9-fold over-expression).

### *Gene amplification*

A total of 50 colon carcinomas were examined for amplification of the c-*myc* cellular proto-oncogene by dot blot hybridisation. Of the 50 samples, 12 were Dukes' A, 17 were Dukes' stage B, eight were Dukes' stage C and 13 were Dukes' stage D. No tumour was found to contain amplified c-*myc*, as compared against hybridisation of β-actin, c-Ki-*ras* exon 1 and carboxypeptidase H (Manser *et al.*, 1990), a gene known to be present as a single copy in the human genome (DRS, unpublished data), see Figure 2 for representative dot blots. In confirmation of the dot blot analysis, no amplification could be discerned in ten samples analysed by genomic Southern blotting. In this case, 10 µg of DNA from the tumour and 10 µg of DNA from matched normal mucosa of the same patient was digested with the restriction endonuclease Eco RI, the digest products were separated on a 0.8% agarose gel and then transferred to solid nylon matrix support prior to hybridisation with a c-*myc* cDNA probe. Results shown in Figure 3 show only the expected single band at 12.5 kb (Taub *et al.*, 1982; Dalla Favera *et al.*, 1983) which is of equal intensity between the tumour sample and the normal mucosa. No amplification is therefore present. Furthermore no rearrangement of the c-*myc* gene was found at this level of resolution in any of the ten samples.

### *Gene rearrangements*

The Southern analysis in Figure 3 shows that of the ten samples analysed no detectable rearrangements were found. For this reason a larger number of samples was examined. A further 32 samples were digested with the restriction endonuclease *Eco*R1 (in this case only DNA from the tumour was examined) and the DNA separated by agarose gel electrophoresis. The final sample composition was 11 Dukes' A tumours, 13 Dukes' B tumours, five Dukes' C tumours and 13 Dukes' D tumours. After Southern transfer, rearrangements of the c-*myc* proto-oncogene were analysed by hybridisation with a c-*myc* cDNA probe. Representative results are shown in Figure 4. As can be seen at this level of resolution no gross rearrangement was found in any of the samples analysed.

### Discussion

The c-*myc* proto-oncogene is the cellular homologue of the v-*myc* oncogene of avian myelocytomatosis virus (Venstrom *et al.*, 1982; Dalla Favera, 1982*b*; Watt *et al.*, 1983*a*,*b*) and is a member of the *myc* family of oncogenes, which contains five other members besides c-*myc*; namely N-*myc*, L*myc*, R*myc*, P*myc* and B*myc* (De Pinho *et al.*, 1987; Ingvarsson *et al.*, 1988). The c-*myc* proto-oncogene is present as a single copy gene in the normal human genome and has been localised to chromosome 8 (specifically at 8q24) (Taub *et al.*, 1982; Dalla Favera *et al.*, 1982*a*; Neel *et al.*, 1982), and consists of one non-coding exon and two coding exons separated by two introns (Hamelyn & Rabbitts, 1983; Watt *et al.*, 1983*a*,*b*; De Pinho *et al.*, 1987).

We have examined the expression of c-*myc* in colorectal tumours. We have found that 66% of colorectal tumours show some degree of over-expression of c-*myc* RNA. The samples analysed were representative of the stages of colorectal tumour as determined by histopathologic analysis. The tumour samples analysed consisted of 25 Dukes' A tumours,

**Figure 1** Representative Northern blot analysis of 21 carcinomas (T) and their corresponding normal mucosa (M) hybridised initially with c-*myc* cDNA and then β-actin and shown as a composite. The number above each pair (T,M) corresponds to a patient.

**Figure 2** Representative dot blot hybridisation of DNA from 16 tumour samples. Each dot contains 10 μg of DNA and filter was hybridised sequentially with probes for c-*myc*, carboxypeptidase H, c-Ki-*ras* and β-actin. Numbers correspond to patients.

25 Dukes' B tumours, 25 Dukes' C and 25 Dukes' D tumours. The degree of over-expression does not correlate with either stage of the tumour as is shown in Table II (although in agreement with other workers (Finley *et al.*, 1989) there is perhaps a slight reduction of the percentage of late state tumours over-expressing c-*myc* i.e. Dukes' stage C and D tumours, although as with Finley *et al.*, this reduction is not statistically significant), or with the age or sex of the donor (Table IV and V respectively). The degree of over-expression ranges from 5-fold to in excess of 400-fold over-expression, but again this does not correlate with the stage of the tumour (Table III).

Rothberg *et al.* (1985), found a significant correlation between the site of the tumour and the over-expression of c-*myc* in a study on 38 colorectal tumours. In their study they find that 81% of left side tumours (those of the rectum, splenic fixture, sigmoid colon and descending colon) over-express c-*myc*, whereas only 36% of right side tumours (those of the caecum, hepatic flexure, ascending colon and transverse colon) show elevated levels of c-*myc* expression (or alternatively 85% of elevated c-*myc* expression is found in left side tumours, whereas only 15% of elevated expression is found in right side tumours). In our study, however, we find that an almost identical proportion of left and right side tumours over-express c-*myc*, i.e. 65% and 60% respectively, although it should be noted that 80% of the tumours in our sample cohort are left side tumours, whereas only 15% of the tumours are right side tumours (five tumours are not included in this analysis as the donors had multiple colorectal tumours). Nevertheless our sample size is more than 2.5 times the size of that used in the study by Rothberg *et al.*

A significant correlation was found in our study between c-*myc* being over-expressed in the tumour and the presence of synchronous polyps elsewhere in the colon (Table VII). Synchronous polyps were found in 24 of the patients. Twenty-two of these cases occurred in patients with c-*myc* over-expression in the tumour, whereas only two of the cases of synchronous polyps were found in those patients whose tumours did not over-express c-*myc*. This correlation is statistically significant ($P < 0.01$). Furthermore the fact that

**Figure 3** Representative Southern hybridisation analysis of c-*myc* in seven carcinomas (T) and compared with their corresponding normal mucosa (M). Each lane consists of 10 μg of DNA digested with *Eco*R1. The 12.5 Kb c-*myc* band is indicated. Band size is estimated against λ DNA digested with *Hind*III (Lane MA) and sized as shown. The number above each pair of lanes corresponds to the patient number.

**Figure 4** Representative Southern analysis of c-*myc* gene rearrangement in 20 carcinoma samples. Numbers above lanes correspond to patient numbers. Each lane contains approximately 10 µg of DNA digested with *Eco*R1. Indicated is the 12.5 Kb c-*myc* product as compared with λ DNA digested with *Hind*III (Lane MA, size of bands shown).

5% of the patients in this study showed synchronous cancers and that 80% of these patients showed c-*myc* over-expression in the main tumour, might indicate that c-*myc* changes are more widespread throughout the colon than has been found with other oncogenes and tumour suppressor genes such as c-Ki-*ras* and p53, whose changes tend to be localised to the site of the tumour. It is possible to speculate therefore that a wide spread colonic alteration could be genetically predetermined. This supposition can be partially supported by the wide range of basal levels of c-*myc* messenger RNA noted by ourselves (data not shown) and others (Finley *et al.*, 1989).

Amplification and rearrangement of the c-*myc* cellular proto-oncogene has been shown to be associated with several different malignancies (Yokota *et al.*, 1986; Little *et al.*, 1983; Ocadiz *et al.*, 1987; Asker *et al.*, 1988; Alitalo *et al.*, 1983; Kozbar & Croce, 1984; Collins & Groudine, 1982; McCarthy *et al.*, 1984; Dalla Favera *et al.*, 1982*c*; Lu *et al.*, 1988; Rothberg *et al.*, 1984; Nakasato *et al.*, 1984; Heerdt *et al.*, 1991). However, we have examined 50 colorectal tumours of different stages and find no evidence of gene amplification. Furthermore, examination of some 42 tumours by genomic Southern analysis shows no evidence of gene rearrangements. The lack of amplification or rearrangement of the c-*myc* gene in colorectal carcinomas clearly indicate that a different mechanism of activation is occurring in these tumours as opposed to tumours of the lymphatic system such as Burkitt's lymphoma, and tumours derived from uterine cervix (Ocadiz *et al.*, 1987), esophageal cancers (Lu *et al.*, 1988), hematopoietic malignancies (Rothberg *et al.*, 1984) and stomach cancers (Nakasato *et al.*, 1984), where amplification and/or rearrangement have been shown to be correlated with tumorigenesis in primary biopsy samples.

Hence, in colorectal cancers as activation of c-*myc* is not a result of either amplification or rearrangement then activation could result from either point mutations in the c-*myc* gene, either in the promoter region or within the first exon as has been shown for some other malignancies, or possibly by the activation or deactivation of a trans-activating factor. This latter possibility is supported by studies that show that c-*myc* over-expression is correlated with loss of chromosome 5 alleles (Erisman *et al.*, 1989). Furthermore the introduction of chromosome 5 by microcell fusion into colon carcinoma cell lines leads to the suppression of c-*myc* deregulation (Rodriguez-Alfageme *et al.*, 1992), possibly by the reintroduction of a functional APC gene, a gene that is implicated in the genesis of spontaneous colorectal cancers and highly implicated in the familial adenomatous polyposis syndrome, an inherited susceptibility to colon cancer and which is known to reside on chromosome 5 (Groden *et al.*, 1991; Joslyn *et al.*, 1991).

We are grateful to Ms Janice Wong for providing clinical data, Dr Jean Ho for the histopathology and Dr David Murphy, IMCB, Singapore for critical reading of this manuscript. This work was supported by grants from the Singapore Totalisator Board, the Lee Foundation and the Shaw Foundation, Singapore.

### References

ALEXANDER, R.J., BUXBAUM, J.N. & RAICHT, R.F. (1986). Oncogene alterations in primary human colon tumors. *Gastroenterology*, **91**, 1503–1510.

ALITALO, K., SCHWAB, M., LIN, C.C., VARMUS, H.E. & BISHOP, J.M. (1983). Homogeneously staining chromosomal regions contain amplified copies of an abundantly expressed cellular oncogene (c-*myc*) in malignant neuroendocrine cells from a human colon carcinoma. *Proc. Natl Acad. Sci. USA*, **80**, 1707–1711.

ASKER, C., MARENI, C., COVIELLO, D., INGVARSSON, S., SESSAREGO, M., ORIGONE, P., KLEIN, G. & SUMEIGI, J. (1988). Amplification of c-*myc* and pvt-1 homologous sequences in acute nonlymphatic leukaemia. *Leuk. Res.*, **12**, 523–527.

BAKER, S.J., FEARON, E.R., NIGRO, J.M., HAMILTON, S.R., PREISINGER, A.C., JESSUP, J.M., VANTUINEN, P., LEDBETTER, D.H., BARKER, D.F., NAKAMURA, Y., WHITE, R. & VOGELSTEIN, B. (1989). Chromosome 17 deletions and p53 gene mutations in colorectal carcinomas. *Science*, **244**, 217–220.

BAKER, S.J., PREISINGER, A.C., JESSUP, J.M., PARASKEVA, C., MARKOWITZ, S., WILSON, J.K.V., HAMILTON, S. & VOGELSTEIN, B. (1990). p53 gene mutations occur in combination with 17p allelic deletions as late events in colorectal tumorigenesis. *Cancer Res.*, **50**, 7717–7722.

BARBACID, M. (1987). *ras* genes. *Annu. Rev. Biochem.*, **56**, 779–827.

BOS, J.L. (1989). *ras* oncogenes in human cancer: A review. *Cancer Res.*, **49**, 4682–4689.

BOS, J.L., FEARON, E.R., HAMILTON, S.R., VERLAAN-DE VRIES, M., VAN BOOM, J.H., VAN DER EB, A.J. & VOGELSTEIN, B. (1987). Prevalence of *ras* gene mutations in human colorectal cancers. *Nature*, **327**, 293–279.

BURMER, G.C. & LOEB, L.A. (1989). Mutations in the KRAS2 oncogene during progressive stages of human colon carcinoma. *Proc. Natl Acad. Sci. USA*, **86**, 2403–2407.

BURMER, G.C., LEVINE, D.S., KULANDER, B.G., HAGGITT, R.C., RUBIN, C.E. & RABINOVITCH, P.S. (1990). c-Ki-*ras* mutations in chronic ulcerative colitis and sporadic colon carcinoma. *Gastroenterology*, **99**, 416–420.

CALABRETTA, B., KACZMAREK, L., MING, P.-M.L., AU, F. & MING, S.-C. (1985). Expression of c-myc and other cell cycle dependent genes in human colon neoplasia. *Cancer Res.*, **45**, 6000–6004.

CHOMCZNSKI, P. & SACCHI, N. (1987). Single-step method of RNA isolation by acid guanidinium thiocyanate-phenol-chloroform extraction. *Anal. Biochem.*, **162**, 156–159.

COLLINS, S. & GROUDINE, M. (1982). Amplification of endogenous *myc*-related sequences in a human myeloid leukaemia cell line. *Nature*, **298**, 679–681.

DALLA FAVERA, R., BREGNI, M., ERIKSON, J., PATTERSON, D., GALLO, R.C. & CROCE, C.M. (1982*a*). Assignment of the human c-*myc* onc-gene to the region of chromosome 8 which is translocated in Burkitt lymphoma cells. *Proc. Natl Acad. Sci. USA*, **79**, 7824–7827.

DALLA FAVERA, R., GELMANN, E.R., MARTINOTTI, S., FRANCHINI, G., PAPAS, T.S., GALLO, R.C. & WONG-STAAL, F. (1982*b*). Cloning and characterisation of different sequences related to the onc gene (v-*myc*) of avian myelocytomatosis virus (MC29). *Proc. Natl Acad. Sci. USA*, **79**, 6497–6501.

DALLA FAVERA, R., WONG-STAAL, F. & GALLO, R.C. (1982*c*). onc gene amplification in promyelotic leukaemia cell line HL-60 and primary leukaemic cells of the same patient. *Nature*, **299**, 61–63.

DALLA FAVERA, R., MARTINITTI, S., GALLO, R.C., ERIKSON, J. & CROCE, C.M. (1983). Translocation and rearrangements of the c-*myc* onc-gene in human undifferentiated B-cell lymphomas. *Science*, **219**, 963–967.

DAVIS, L.G., DIBNER, M.D. & BATTEY, J.F. (1986). Preparation of DNA from eukaryotic cells. In *Basic Methods in Molecular Biology*. Elsevier.

DE PINHO, R.A., MITSOCK, L., HATTON, K., FERRIER, P., ZIMMERMAN, K., LEGOUY, E., TESFAYE, A., COLLUM, R., YANCOPOULOS, G., NISEN, P., KRIZ, R. & ALT, F. (1987). *myc* family of cellular oncogenes. *J. Cell Biochem.*, **33**, 257–266.

EICK, D., PIECHACZYK, M., HENGLEIN, B., BLANCHARD, J.M., TRAUB, B., KOFLER, E., WIEST, S., LENOIR, G.M. & BORNKAMM, W. (1985). Aberrant c-*myc* RNAs of Burkitts lymphoma cells have longer half lives. *EMBO J.*, **4**, 3717–3725.

ERISMAN, M.D., ROTHBERG, P.G., DIEHL, R.E., MORSE, C.C., SPANDORFER, J.M. & ASTRIN, S.M. (1985). Deregulation of c-*myc* gene expression in human colon carcinoma is not accompanied by amplification or rearrangement of the gene. *Mol. Cell Biol.*, **5**, 1969–1976.

ERISMAN, M.D., LITWIN, S., KEIDAN, R.D., COMIS, R.L. & ASTRIN, S.M. (1988). Noncorrelation of the expression of the c-*myc* oncogene in colorectal carcinoma with recurrence of disease or patient survival. *Cancer Res.*, **48**, 1350–1355.

ERISMAN, M.D., SCOTT, J.K. & ASTRIN, S.M. (1989). Evidence that the familial adenomatous polyposis gene is involved in a subset of colon cancers with a complementable defect in c-*myc* regulation. *Proc. Natl Acad. Sci. USA*, **86**, 4264–4268.

FEARON, E.R. & JONES, P.A. (1992). Progressing towards a molecular description of colorectal cancer development. *FASEB J.*, **6**, 2783–2790.

FEARON, E.R. & VOGELSTEIN, B. (1990). A genetic model for colorectal tumorigenesis. *Cell*, **61**, 759–767.

FEINBERG, A.P. & VOGELSTEIN, B. (1983). A technique for radiolabelling DNA restriction endonuclease fragments to high specific activity. *Anal. Biochem.*, **132**, 6–13.

FEINBERG, A.P. & VOGELSTEIN, B. (1984). A technique for radiolabelling DNA restriction endonuclease fragments to high specific activity. *Addendum Anal. Biochem.*, **137**, 266–267.

FINLEY, G.G., SCHULZ, N.T., HILL, S.A., GEISER, J.R., PIPAS, J.M. & MEISLER, A.I. (1989). Expression of the *myc* gene family in different stages of human colorectal cancer. *Oncogene*, **4**, 963–971.

FORRESTER, K., ALMOGUERA, C., HAN, C., GRIZZLE, W.E. & PERUCHO, M. (1987). Detection of high incidence of K-*ras* oncogenes during human tumorigenesis. *Nature*, **327**, 298–303.

GRAND, R.J.A. & OWENS, D. (1991). The biochemistry of *ras* p21. *Biochem. J.*, **279**, 609–631.

GRODEN, J., THLIVERIS, A., SAMOWITZ, W., CARLSON, M., GELBERT, L., ALBERTSEN, H., JOSLYN, G., STEVENS, J., SPIRIO, L., ROBERTSON, M., SARGEANT, L., KRAPCHO, K., WOLFF, E., BURT, R., HUGHES, J.P., WARRINGTON, J., MCPHERSON, J., WASMUTH, J., LE PASLIER, D., ABDERRAHIM, H., COHEN, D., LEPPERT, M. & WHITE, R. (1991). Identification and characterisation of the familial adenomatous polyposis gene. *Cell*, **66**, 589–600.

HAMELYN, P.H. & RABBITTS, T.H. (1983). Translocation joins c-*myc* and immunoglobulin G1 genes in a Burkitt lymphoma revealing a third exon in c-*myc* oncogene. *Nature*, **304**, 135–139.

HEERDT, B.G., MOLINAS, S., DEITCH, D. & AUGENLICHT, L.H. (1991). Aggressive subtypes of human colorectal tumours frequently exhibit amplification of the c-*myc* gene. *Oncogene*, **6**, 125–129.

HOLLSTEIN, M., SIDRANSKY, D., VOGELSTEIN, B. & HARRIS, C.C. (1991). p53 mutations in human cancers. *Science*, **253**, 49–53.

IMASEKI, H., HAYASHI, H., TAIRA, M., ITO, Y., TABATA, Y., ONODA, S., ISONO, K. & TATIBANA, M. (1989). Expression of c-*myc* oncogene in colorectal polyps as a biological marker for monitoring malignant potential. *Cancer*, **64**, 704–709.

INGVARSSON, S., ASKER, C., AXELSON, H., KLEIN, G. & SUMEGI, J. (1988). Structure and expression of B-*myc* a new member of the *myc* gene family. *Mol. Cell Biol.*, **8**, 3168–3174.

JOSLYN, G., CARISON, M., THLIVERIS, A., ALBERTSEN, H., GELBERT, L., SAMOWITZ, W., GRODEN, J., SIEVENS, J., SPIRO, L., ROBERTSON, M., SARGEANT, L., KRAPCHO, K., WOLFF, E., BURT, R., HUGHES, J.P., WARRINGTON, J., MCPHERSON, J., WASMUTH, J., LE PASLIER, D., ABDERRAHIM, H., COHEN, D., LEPPERT, M. & WHITE, R. (1991). Identification of deletion mutations and three new genes at the familial polyposis locus. *Cell*, **66**, 601–613.

KOZBAR, D. & CROCE, C.M. (1984). Amplification of the c-*myc* oncogene in one of five human breast carcinoma cell lines. *Cancer Res.*, **44**, 438–441.

LEDER, P., BATTEY, J., LENOIR, G., MOULDING, C., MURPHY, W., POTTER, H., STEWART, T. & TAUB, R. (1983). Translocations among antibody genes in human cancer. *Science*, **222**, 765–771.

LITTLE, C.D., NAU, M.M., CARNEY, D.N., GAZDAR, A.F. & MINNA, J.D. (1983). Amplification and expression of the c-*myc* oncogene in human lung cancer cell lines. *Nature*, **306**, 194–196.

LU, S.-H., HSIEH, L.-L., LUO, F.-C. & WEINSTEIN, I.B. (1988). Amplification of the EGF receptor and c-*myc* genes in human esophaegeal cancers. *Int. J. Cancer*, **42**, 502–505.

MANSER, E., FERNANDEZ, D., LOO, L., GOH, P.H., MONTFRIES, C., HALL, C. & LIM, L. (1990). Human carboxypeptidase E: isolation and characterisation of the cDNA clone, sequence conservation, expression and processing in vitro. *Biochem. J.*, **265**, 517–525.

MCCARTHY, D.M., RASOOL, F.V., GOLDMAN, J.M., GRAHAM, S.V. & BIRNIE, G.D. (1984). Genomic alterations involving the c-*myc* proto-oncogene locus during the evolution of a case of chronic granulocytic leukaemia. *Lancet*, **2**, 1362–1365.

MELHEM, M.F., MEISLER, A.I., FINLEY, G.G., BRYCE, W.H., JONES, M.O., TRIBBY, I.I., PIPAS, J.M. & KOSKI, R.A. (1992). Distribution of cells expressing *myc* proteins in human colorectal epithelium, polyps and malignant tumors. *Cancer Res.*, **52**, 5853–5864.

MELTZER, S.J., AHNEN, D.J., BATTIFORA, H., YOKATA, J. & CLINE, M.J. (1987). Proto-oncogene abnormalities in colon cancers and adenomatous polyps. *Gastroenterology*, **92**, 1174–1180.

MULLIS, K.B. & FALOONA, F.A. (1987). Specific synthesis of DNA *in vitro* via a polymerase catalysed chain reaction. *Methods Enzymol.*, **155**, 335–350.

NAKASATO, F., SAKAMOTO, H., MORI, M., HAYASHI, K., SHIMATO, Y., NISHI, M., TAKAO, S., NAKATANI, K., TERADA, M. & SUGIMUREA, T. (1984). Amplification of the c-*myc* oncogene in human stomach cancers. *Jpn. J. Cancer Res.*, **75**, 737–742.

NEEL, B.G., JHANWAR, S.C., CHAGANTI, R.S.K. & HAYWARD, W.S. (1982). Two human c-onc genes are located in the long arm of chromosome 8. *Proc. Natl Acad. Sci. USA*, **79**, 7842–7846.

NIGRO, J.M., BAKER, S.J., PREISINGER, A.C., JESSUP, J.M., HOSTETTER, R., CLEARY, K., BIGNER, S.H., DAVIDSON, N., BAYLIN, S., DEVILEE, P., GLOVER, T., COLLINS, F.S., WESTON, A., MODALI, R., HARRIS, C.C. & VOGELSTEIN, B. (1989). Mutations in the p53 gene occur in diverse human tumor types. *Nature*, **342**, 705–708.

OCADIZ, R., SAUCEDA, R., CRUZ, M., GRAEF, A.M. & GARIGLIO, P. (1987). High correlation between molecular alterations of the c-*myc* oncogene and carcinoma of the uterine cervix. *Cancer Res.*, **47**, 4173–4177.

RABBITTS, T.H., HAMLYN, P.H. & BAER, R. (1983). Altered nucleotide sequences of a translocated c-*myc* gene in Burkitt lymphoma. *Nature*, **306**, 760–765.

RABBITTS, T.H., FORSTER, A., HAMLYN, P. & BAER, R. (1984). Effect of somatic mutations within translocated c-*myc* genes in Burkitt's lymphoma. *Nature*, **309**, 592–597.

RODRIGUEZ, N.R., ROWAN, A., SMITH, M.E.F., KERR, I.B., BODMER, W.F., GANNON, J.V. & LANE, D.P. (1990). p53 mutations in colorectal cancer. *Proc. Natl Acad. Sci. USA*, **87**, 7555–7559.

RODRIGUEZ-ALFAGEME, C., STANBRIDGE, E.J. & ASTRIN, S.M. (1992). Suppression of deregulated c-*MYC* expression in human colon carcinoma cells by chromosome 5 transfer. *Proc. Natl Acad. Sci. USA*, **89**, 1482–1486.

ROTHBERG, P.G., ERISMAN, M.D., DIEHL, R.E., ROVIGATTI, U.G. & ASTRIN, S.M. (1984). Structure and expression of the oncogene c-*myc* in fresh tumor material from patients with hematopoietic malignancies. *Mol. Cell Biol.*, **4**, 1096–1103.

ROTHBERG, P.G., SPANDORFER, J.M., ERISMAN, M.D., STAROSCIK, R.N., SEARS, H.F., PETERSON, R.O. & ASTRIN, S.M. (1985). Evidence that c-*myc* expression defines two genetically distinct forms of colorectal adenocarcinoma. *Br. J. Cancer*, **52**, 629–632.

SAIKI, R.K., SCHARF, S.J., FALLOONA, F., MULLIS, K.B., HOM, G.T., ERLICH, H.A. & AENHEIM, N. (1985). Enzymatic amplification of β-globin genomic sequences and restriction site analysis for diagnosis of sickle cell anemia. *Science*, **230**, 1350–1354.

SAIKI, R.K., GELFAND, D.H., STOFFEL, S., SCHARF, S.J., HIGUCHI, R., HOM, G.T., MULLIS, K.B. & ERLICH, H.A. (1988). Primer-directed enzymatic amplification of DNA with a thermostable DNA polymerase. *Science*, **239**, 487–491.

SAMBROOK, J., FRITSCH, E.F. & MANIATIS, T. (1989). *Molecular Cloning: A Laboratory Manual.* Second Edition. Cold Spring Harbor Laboratory Press, USA.

SIKORA, K., CHAN, S., EVAN, G., GABRA, H., MARKHAM, N., STEWART, J. & WATSON, J. (1987). c-*myc* oncogene expression in colorectal cancer. *Cancer*, **59**, 1289–1295.

STEWART, J., EVAN, G., WATSON, J. & SIKORA, K. (1986). Detection of the c-*myc* oncogene product in colonic polyps and adenocarcinomas. *Br. J. Cancer*, **53**, 1–6.

TAUB, R., KIRSCH, I., MORTON, C., LENOIR, G., SWAN, D., TRONICK, S., AARONSON, S. & LEDER, P. (1982). Translocation of the c-*myc* gene into the immunoglobulin heavy chain locus in human Burkitt lymphoma and murine plasmacytoma cells. *Proc. Natl Acad. Sci. USA*, **79**, 7837–7841.

TSUBOI, K., HIRAYOSHI, K., TAKEUCHI, K., SABE, H., SHIMADA, Y., OHSHIO, G., TOBE, T. & HATANAKA, M. (1987). Expression of the c-*myc* gene in human gastrointestinal malignancies. *Biochem. Biophys. Res. Comm.*, **146**, 699–704.

VENSTROM, B., SHEINESS, D., ZABIELSKI, J. & BISHOP, J. (1982). Isolation and characterisation of c-*myc*, a cellular homologue of the oncogene (v-*myc*) of avian myelocytomatosis virus, strain 29. *J. Virol.*, **42**, 773–779.

VOGELSTEIN, B., FEARON, E.R., KERN, S.E., PREISINGER, A.C., LEPPERT, M., NAKAMURA, Y., WHITE, R., SMITS, A.M.M. & BOS, J.L. (1988). Genetic alterations during colorectal tumor development. *N. Engl. J. Med.*, **319**, 525–532.

WATT, R., NISHIKURA, K., SORRENTINO, J., AR-RUSHDI, A., CROCE, C.M. & ROVERA, G. (1983*a*). The structure and nucleotide sequence of the 5′ end of the human c-*myc* oncogene. *Proc. Natl Acad. Sci. USA*, **80**, 6307–6311.

WATT, R., STANTON, L.W., MARCU, K.B., GALLO, R.C., CROCE, C.M. & ROVERA, G. (1983*b*). Nucleotide sequence of cloned cDNA of human c-*myc* oncogene. *Nature*, **303**, 725–728.

YOKATA, J., TSUNETSUGA-YOKOTO, Y., BATTIFORA, H., LEFEVRE, C. & CLINE, M.J. (1986). Alterations of *myc*, myb and Ha-ras proto-oncogenes in cancers are frequent and show clinical correlation. *Science*, **231**, 261–265.